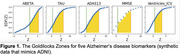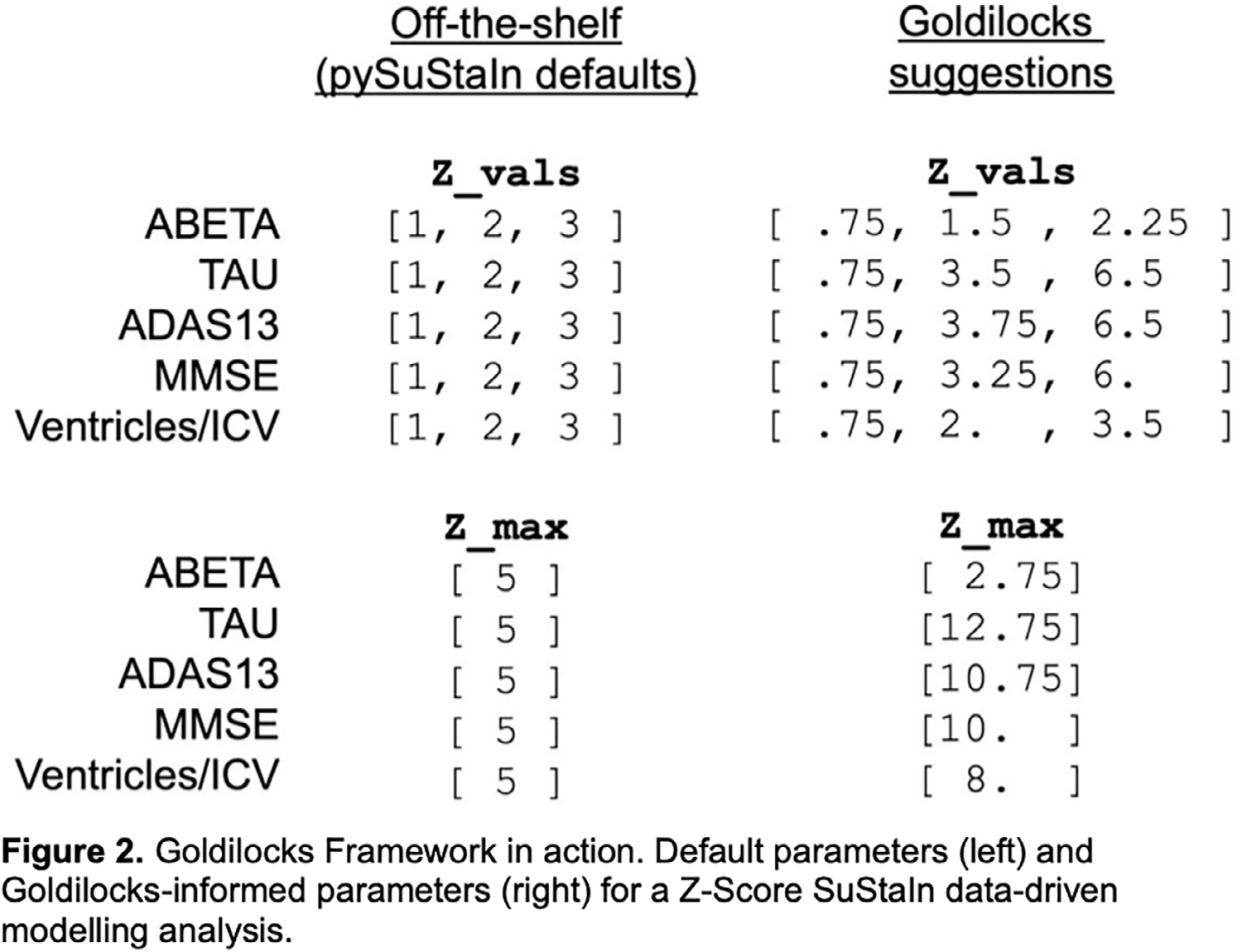# Goldilocks Framework: semi‐automated model configuration for democratising data‐driven analyses of Alzheimer’s disease progression

**DOI:** 10.1002/alz.092363

**Published:** 2025-01-09

**Authors:** Neil P Oxtoby

**Affiliations:** ^1^ University College London, London UK

## Abstract

**Background:**

Data‐driven models are at the mercy of the data upon which they are built. Poorly configured models can lead to misleading conclusions. Here we introduce a framework called Goldilocks for carefully configuring data‐driven modelling analyses to remain in the Goldilocks Zone — features that are not too weak (in terms of disease signal), and model (hyper)parameters that are not too strong (with respect to available training data).

**Method:**

Goldilocks has three key steps: 1. Normalise data. Transform data from cases (e.g., patients) into “disease signal”, e.g., normative z‐scores relative to controls. 2. Feature selection. Identify candidate input features having the necessary minimum signal. 3. Model configuration (hyperparameters). Remain within the available maximum signal.

Experiments: we synthesize ADNI‐like biomarker data, define the Goldilocks Zone to lie between two event horizons (minimum and maximum disease signal), and demonstrate Goldilocks for configuring a Subtype and Stage Inference (SuStaIn) analysis. Minimum disease signal z_min_ is determined by the tipping point between case/control data, where the half‐normal cumulative density function HN‐CDF(z_min_)==0.5. Maximum disease signal z_max_ is defined where the empirical distribution function EDF(z_max_)==0.9, such that 10% of the biomarker’s data (from cases, not controls) exceeds z_max_.

**Result:**

We synthesized z‐scored ADNI‐like synthetic CSF biomarkers (ABETA, TAU), cognitive test scores (ADAS13, MMSE), and a brain volume (Ventricles normalised by intracranial volume). Figure 1 shows biomarker empirical distributions as a function of z‐score, and the Goldilocks Zone between event horizons at z_min_==0.67 and EDF(z_max_)==0.9. Since EDF(z_min_) < 0.5 for all biomarkers, they each contain sufficient disease signal to be included in a data‐driven modelling analysis like SuStaIn. The maximum disease signal for each biomarker ranged between approximately 2 to 6. Figure 2 compares default parameters in a Z‐Score SuStaIn analysis (using the official pySuStaIn software) to Goldilocks‐informed parameters.

**Conclusion:**

Goldilocks has the potential to improve validity and interpretability of data‐driven discoveries in general while also making data‐driven modelling accessible to non‐experts. This promotes democratization of data‐driven analyses, unlocking previously untapped data responsibly. This is particularly important in fields such as computational medicine, where data‐driven decisions can relate to life‐or‐death outcomes.